# A cross-sectional study on the association between executive functions and social disabilities in people with a psychotic disorder

**DOI:** 10.1016/j.scog.2025.100349

**Published:** 2025-02-12

**Authors:** B.C. van Aken, R. Rietveld, A.I. Wierdsma, Y. Voskes, G.H.M. Pijnenborg, J. van Weeghel, C.L. Mulder

**Affiliations:** a Department of Psychiatry, Erasmus MC, Epidemiological and Social Psychiatric Research Institute, Rotterdam, the Netherlands; b Fivoor Forensic Psychiatric Institute, Rotterdam, the Netherlands; c Department of Ethics, Law and Humanities, Amsterdam UMC, Amsterdam, the Netherlands; d GGz Breburg, Tilburg, the Netherlands; e Department of Psychotic Disorders, GGZ Drenthe, Assen, the Netherlands; f Department of Clinical and Developmental Neuropsychology, Faculty of Behavioural and Social Sciences, University of Groningen, Groningen, the Netherlands; g Phrenos Centre of Expertise, Utrecht, the Netherlands; h Tranzo Department, Tilburg School of Behavioural and Social Sciences, Tilburg University, Tilburg, the Netherlands; i Bavo-Europoort Mental Health Care, Rotterdam, the Netherlands

**Keywords:** Social recovery, Role salience, Role difficulty, Functional recovery, Executive functioning, Employment status

## Abstract

**Background:**

Social recovery (SR) in people with psychotic disorders involves taking on social roles and completing daily tasks. Functional recovery (FR), particularly executive functions, is critical for these roles. Psychotic disorder patients often experience severe cognitive impairments, especially in executive functions. This study investigates the relationship between functional and social recovery in individuals with psychotic disorders and examines the effect of employment status on this association.

**Method:**

This cross-sectional study involved people with a psychotic disorder. SR was measured using the WHO-DAS, divided into Daily Functioning (DF) and Social Functioning (SF) scales. FR was measured using the BRIEF-A and the TOL. Employment status was categorized into Non-active, Otherwise Active, and Active. The Likelihood-ratio Test (LRT) was used for model selection.

**Results:**

Data from 251 participants (mean age 41.5) showed that the BRIEF-A affected both DF and SF, while the TOL only affected DF. Only being Otherwise Active influenced DF. Employment status had no influence on SF. Being Otherwise Active positively influenced perceived disabilities in Daily Functioning.

**Conclusion:**

Measuring executive functions using both performance-based and self-report measures is important. Both measures are associated differently with perceived disabilities in daily and social functioning. Being a volunteer or looking for a job positively influences perceived disabilities in Daily Functioning.

## 1 Introduction

In people with a psychotic disorder, recovery is a multidimensional concept. One way to operationalize this multidimensional concept is to divide it into personal, symptomatic, functional, and social recovery (van der Stel, 2012).

Functional recovery entails a person's ability ‘to recover or compensate for the loss of skills and impairments in cognitive functioning’ (van der Stel, 2012; van Aken et al., 2021; Mulder et al., 2021). As cognitive impairments are thought to be a core feature of psychotic disorders (Velligan and Bow-Thomas, 1999; Kahn and Keefe, 2013; Keefe and Harvey, 2012; Holmén et al., 2012), recovery from them is now seen as a separate dimension of recovery (Stel JCvd., 2015; Association AP and Association AP, 2013; Tandon et al., 2013). Since executive functions – which include goal-directed processes involving active real-time decision-making, planning, self-control and initiating behaviour (Frangou, 2010; Coulacoglou and Saklofske, 2017; Kerns et al., 2008; van der Stel et al., 2015) – are one of the cognitive skills that are the most impaired (Meier et al., 2014; Liu et al., 2011; Chan et al., 2012). The fact that they are diminished before the onset of the disorder (Mollon and Reichenberg, 2018; Dickson et al., 2012), is associated with the risk of developing a psychosis (Poletti et al., 2019). The impairments in question stabilize after the first episode of psychosis (Fucetola et al., 2000; Bowie et al., 2008; Kurtz, 2005; Zabala et al., 2010; Rabinowitz et al., 2002; Sheffield et al., 2018; Tempelaar et al., 2017; Barder et al., 2013; Freedman and Brown, 2011). During the illness, they negatively affect daily functioning, career, education, social relationships, and community functioning (Jones and Harvey, 2020; McGurk et al., 2003; Barkley, 2012; Bowie et al., 2006; Bowie et al., 2010; Green et al., 2004; Ventura et al., 2009; Muralidharan et al., 2020; Niendam et al., 2007). Due to the severe impact of these functions, they are used here as a (limited) way to operationalize functional recovery (van Aken et al., 2021; van Aken et al., 2022).

Social recovery is defined as ‘establishing and maintaining rewarding relationships with family, friends, peers, colleagues and significant others’ (Whitley and Drake, 2010). It entails both daily activities like household chores, but also activities such as participation in society. It is about the discovery and perception of the different social roles a person can maintain (Wierdsma et al., 2023), as well as the difficulties they encounter in these roles (Classifications: WHO Disability Assessment Schedule 2.0 WHODAS 2.0, 2014). Although people are inherently social creatures, with a fundamental need to be socially accepted (Baumeister and Leary, 1995), some subgroups have different baseline levels of disability and different recovery pathways (Hodgekins et al., 2015). These differences in social abilities, disabilities and roles may help us understand processes of social recovery, which, until now, have been thought to be relatively stable over time, but also to be low for those with severe symptoms and an early age of onset (Hodgekins et al., 2015). In this paper, social recovery is operationalized using the daily and social functioning scale of the WHODAS 2.0, which is derived from International Classification of Functioning, Disability and Health (ICF) model (Guilera et al., 2012; Chwastiak and Von Korff, 2003).

The link between daily and social functioning and executive functioning is a complex one. Impairments in skills such as cognition and executive functions are thought to be key predictors of greater exclusion from social and occupational roles (Green, 1996; Robinson et al., 2004; Carter, 2006). In turn, the lack of a social or occupational role increases problems with inhibitory control (Xu et al., 2017; Xu et al., 2016), working memory (Xu et al., 2018) and impulsive and aggressive behaviour (Lurquin et al., 2014; Otten and Jonas, 2013). A study by Chen (55) found that cognition improved more over time in schizophrenia patients who were employed than in those who were unemployed. These problems can then lead to more severe problems with social functioning (Green et al., 2004; Green, 1996; Green et al., 2000), which lead to worse cognitive problems and vice versa. It thus seems to be a vicious cycle. One aspect within the association between having work (or not) and cognitive functioning is the total hours worked per week. A study by Van Duin (Van Duin et al., 2021) found that an increase in the total number of hours worked was associated over time with small improvements in processing speed and working memory (Van Duin et al., 2021). However, other studies showed that an increase in the total number of hours worked was associated with a decline in attention, working memory and visual organization and memory (Karambelas et al., 2018). The association between total hours worked and factors such as working memory, and possibly even executive functions in general, thus remains unclear.

Executive functions can be measured using different forms of measurement, i.e. performance-based measures (Gohier et al., 2009), or using self-report measures (Gioia et al., 2008; Payne et al., 2011). Although both have been validated to measure executive functions, several studies found no correlation (Meltzer et al., 2017; Nordvall et al., 2017). For social and daily functioning, as well as executive functions, it is therefore important to broaden our view of the constructs and the association between them.

The aim of this study was to investigate the association between social and functional recovery. For social recovery, we examined limitations in daily functioning and social functioning, based on the two dimensions of disability of the WHODAS 2.0; for functional recovery, we examined executive functioning. To do so, we tested executive functioning in two ways by using a performance-based test and a self-report questionnaire, thereby following the recommendation that multiple forms of testing should be used (Isquith et al., 2013; Goldstein et al., 1993; Prouteau et al., 2004). Performance-based testing is thought to reflect a person's technical efficiency (e.g., what you are capable of in a setting with almost no distractions). Self-report testing is thought to reflect a person's ability in an environmental context (e.g. what you are capable of in a setting with ongoing distractions) (Bowie and Harvey, 2006; Weyandt et al., 2014). Since Daily Functioning – such as self-care – often involves activities that are done within a familiar environment, allowing for focused attention with few distractions (Kulmala et al., 2019), we hypothesized that performance-based testing would be associated with disabilities in daily functioning. Social Functioning on the other hand, often features multiple people, resulting in a more demanding and unpredictable setting with multiple distractions (Sveen et al., 1999). Therefore, we hypothesized that the self-report questionnaire would be associated with disabilities in social functioning.

Finally, since studies show that cognition improved more over time for those employed (Chen et al., 2020), we included employment status for examining its (interaction) effect on the association between executive function and social recovery. We hypothesized that employment status would have an effect on the association between executive functions and daily and social functioning.

## 2 Methods

The UP'S study is a 10-year observational cohort study examining recovery processes in individuals with psychotic disorders (van Aken et al., 2021; Mulder et al., 2021). Conducted in collaboration with nine mental healthcare institutes in the Southwest of the Netherlands and the Erasmus University Medical Centre, it involves clients of participating community mental healthcare teams with similar ways of care deliverance (mainly certified Fast Assertive Community Treatment (FACT) teams) of the institutions. These clients meet specific inclusion criteria: a primary DSM-5 diagnosis within the schizophrenia spectrum (e.g., schizophrenia, schizoaffective disorder, psychotic disorder NOS) and an age range of 18–65. Non-Dutch speakers are excluded due to the study's reliance on Dutch-language questionnaires.

Researchers, supported by trained students, handle recruitment, interviews, and follow-ups at mental health care teams, community sites, patients' homes, or at the Erasmus University Medical Centre. For each participating team, 30 eligible participants are randomly selected from anonymized electronic patient files (EPFs). If there are fewer than 30 patients in a team that meet the inclusion criteria, all eligible individuals may be invited to participate unless they cannot be approached due to issues like active psychosis or relocation.

**Fig. 1:**
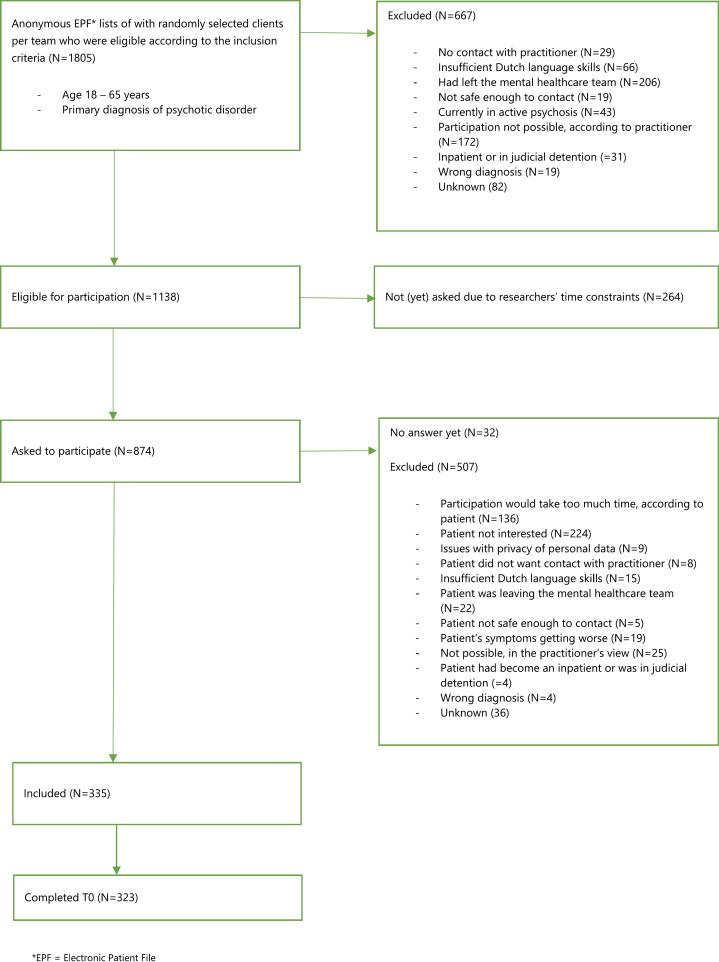
Inclusion flowchart for the UP'S study.

Interested patients receive study details and have two weeks to consider participation, providing written informed consent if they agree. Patients who decline participation are asked for their reason and not contacted again. Unfortunately, no further data were collected on those who declined, limiting comparisons between participants and non-participants. Fig. 1 shows the inclusion flowchart and the number of participants with baseline interview completion. This study includes the data of all those participants.

The study protocol has been approved by the accredited Dutch Medical Ethical Trial Committee (METC) of the Erasmus Medical Centre. Further details on the inclusion process of the UP'S study can be found in van Aken (van Aken et al., 2021).

### 2.1 Questionnaires

#### 2.1.1 Functional recovery

*The Behaviour Rating Inventory of Executive Functioning for Adults (BRIEF-A)* is a 76-item self-report questionnaire assessing executive function in daily life (Roth et al., 2014). Each item is rated from never (van der Stel, 2012) to always (Mulder et al., 2021) and falls under one of nine subscales, organized into either the Behaviour Regulation Index (four subscales) or the Metacognitive Index (five subscales). Together, these indices form the Global Executive Functioning scale. T-scores and percentile scores are calculated, with t-scores of 60–65 indicating subclinical executive functioning, and scores above 65 (or percentiles above 90) indicating clinical impairment. The BRIEF-A includes three validity scales—negativity, infrequency, and inconsistency—where scores above cut-off invalidate the questionnaire, excluding it from further analysis. This measure has been validated in schizophrenia samples (Power et al., 2012), with a Cronbach's α of 0.91 and a MacDonald's Omega of 0.95 in this study.

*The Tower of London (TOL)* is a performance-based planning and executive functioning task containing 20 basic assignments and with 2 bonus items that can be answered if all basic items have been answered correctly, each within 20 s per item. The task consists of two pictures that contain three sticks with three beads (red, blue, and green) stacked on them. The goal is to identify the number of steps needed to go from the first to the second picture (Shallice, 1982).

#### 2.1.2 Social recovery

*The World Health Organization – Disability Assessment Schedule 2.0 (WHODAS)* assesses activity limitations and participation restrictions regardless of diagnosis (Chwastiak and Von Korff, 2003). Based on the International Classification of Functioning, Disability and Health (ICF) model (Guilera et al., 2012; Chwastiak and Von Korff, 2003), it has three versions: a 36-item, 12-item, and 12 + 24-item version. It can be filled in by an interviewer, by a proxy, or as a self-report questionnaire. This study used the 36-item self-report version, covering six domains: understanding & communicating, mobility, self-care, getting along, life activities, and participation. Two subscales can be derived: daily functioning (understanding & communicating, mobility, self-care) and social functioning (getting along, life activities, participation). WHODAS scoring includes a simple sum score or a complex score based on item-response theory (IRT) to weigh items by difficulty (Epping-Jordan et al., 2000), which is used in this study. Scores range from 0 (no disability) to 100 (full disability). For non-working respondents, a 32-item version omits work questions. A previous study by Williams et al. proposed a complex model with a general disability factor plus six factors for non-working and seven for working groups (Williams et al., 2021). However, due to interpretation challenges in regression models, we chose the simpler two-factor model. In this study, Cronbach's α of the WHODAS-DF was α = 0.80 and MacDonald's Omega =0.83. For the WHODAS-SF, Chronbach's α was α = 0.81 and MacDonald's Omega = 0.82.

*Employment status* data was collected through Routine Outcome Monitoring (ROM) interviews in mental healthcare institutions, where each institution chooses its own questionnaire. For employment status, all institutions collected the following work categories for each participant: (van der Stel, 2012) paid work; (van Aken et al., 2021) Social Employment Act work/reintegration; (Mulder et al., 2021) training/education; (Velligan and Bow-Thomas, 1999) (early) retirement; (Kahn and Keefe, 2013) volunteer work; (Keefe and Harvey, 2012) incapacitated; (Holmén et al., 2012) unemployed, not seeking work; and (Stel JCvd., 2015) unemployed, seeking work. Since we did not have hours worked per category, we opted to create three new categories: (van der Stel, 2012) Work – Active (statuses #1–3); (van Aken et al., 2021) Work – Otherwise Active (#5 and 8); and (Mulder et al., 2021) Work – Non-active (#4, 6, and 7).

#### 2.1.3 Symptom severity

*The Positive and Negative Symptom Scale – Remission (PANSS-R)* is a shortened version of the original 30-item PANSS, focusing on eight items: three positive symptoms, three negative symptoms, and two general symptoms (Andreasen et al., 2005). Each item is scored from 1 (absent) to 7 (extreme), reflecting symptom severity and behavioural impact (Kay et al., 1987). Our analysis used both subscale means and the total score. If one item was missing, it was imputed with the mean of the other items; if more than one was missing, scores were not calculated. In this cohort, Cronbach's α was 0.67 and MacDonald's Omega 0.73.

### 2.2 Statistical analysis

Descriptive statistics summarized demographic characteristics and questionnaire scores for both the entire sample as well as only the complete cases, with missing values and outliers handled according to questionnaire standards. Employment status was summarized from eight into three categories: (van der Stel, 2012) Work- Active, (van Aken et al., 2021) Work, − Otherwise active and (Mulder et al., 2021) Work – non-active. Scores for the BRIEF-A and TOL were standardized into z-scores based on the mean, for comparison. Correlations were calculated. Violin plots for employment status were made to look at the differences in disability dispersion between all employment statuses and between de WHODAS-DF and WHODAS-SF for each employment status. Generalized linear models assessed associations between executive functioning and WHODAS-DF and WHODAS-SF, controlling for gender, age, and symptom severity. Models were built with and without interactions between BRIEF-A, TOL, and employment status. For employment status, each level was turned into a dummy. Level 3 – Non-active was used as a baseline in each model. The Likelihood-ratio Test (LRT) was used for model selection using the calculation: LR = 2*(LnL2−LnL1). L1 is the Likelihood Ratio Chi-square for the model with interactions, whereas L2 is the Likelihood Ratio Chi-square for the simple model without the interactions. For the selected models assumptions were checked. Analyses were conducted in SPSS version 27.0.

## 3 Results

### 3.1 Patient characteristics

At the time of writing, the data of 323 participants from UP'S cohort study was available and included in this paper. As 251 of the participants had no missing data, they were considered to be ‘complete cases.’ Table 1 shows the descriptive statistics and mean scores of the baseline data used in this study (1.) for all participants, and (2.) only for the complete cases. For the complete sample, the mean age was 41.5 years (SD = 12.3), while the mean time in care in years was 12.3 years (SD = 10.0); 64.4 % were male; 42.1 % had a primary diagnosis of schizophrenia; 31.9 % had secondary vocational education as their highest level of education; and 56,5 % were single without children. Their mean PANSS-R score was 2.0 (0.8). At the time of inclusion, a small majority of our participants (53 %) did not have a paid job.

**Table 1:** Descriptive statistics.

		Baseline (sample *N* = 323)			Baseline (Complete cases *N* = 251)	
		N (%)	Range	Mean (SD)	N (%)	Mean (SD)
Age			18–65	41.5 (12.3)		41.5 (12.0)
Sex (male)		208 (64.4)			165 (66.0)	
Time in care (in years)			0–37	12.3 (10.0)		12.2 (10.1)
Diagnosis	Schizophrenia	136 (42.1)			155 (45.8)	
	Psychosis NOS	66 (20.4)			53 (21.1)	
	Short-term psychotic disorder	46 (14.2)			40 (15.9)	
	Schizoaffective disorder	27 (8.4)			19 (7.6)	
	Other psychotic disorders	48 (14.9)			24 (9.6)	
Employment	1. Active	72 (25.5)			65 (25.8)	
status	2. Otherwise Active	61 (21.5)			55 (22.5)	
	3. Non-active	150 (53)			126 (51.6)	
Symptoms	PANSS-R total		1–4.5	2.0 (0.8)		2.0 (0.8)
WHO-DAS	Daily Functioning		15.2–76.1	30.3 (13.5)		30.5 (13.5)
	*D1. Understanding and communicating*		*10*–*90*	*31.6 (18.5)*		*31.9 (18.7)*
	*D2. Getting around*		*12.5*–*100*	*26.7 (19.1)*		*26.8 (19.1)*
	*D3. Self-care*		*30*–*80*	*33.7 (8.1)*		*33.7 (8.4)*
	Social Functioning		23.9–87	43.2 (14.6)		43.1 (14.5)
	*D4. Getting along with people*		*33.3*–*100*	*46.7 (15.3)*		*46.4 (15.1)*
	*D5.1 Life activities*		*30*–*100*	*43.5 (20.1)*		*43.3 (20.1)*
	*D6. Participation in society*		*16.7*–*91.7*	*41.6 (18.7)*		*41.4 (18.8)*
Executive Functioning	BRIEF-A⁎		35–83	56.5 (10.3)		56.5 (10.4)
	Tower of London (TOL)		0–22	15.4 (4.8)		15.4 (5.0)

Complete cases are those with no missing data on any of the variables displayed. The total baseline group shows the mean for the entire group including participants who have missing data on any of the data displayed.

⁎ BRIEF-A scores consist only of those having a valid score according to BRIEF-A guidelines.

The mean BRIEF-A score was 56.5 (SD = 10.3), whereas the mean TOL score was 15.4 (4.8). Whereas the mean WHODAS-DF subscale was 30.3 (SD = 13.5), the mean WHODAS-SF subscale was 43.2 (SD = 14.6).

### 3.2 Plots and correlations

As Table 2 shows, the BRIEF-A was correlated with both the WHODAS-DF (*r* = 0.56) and WHODAS-SF scales (*r* = 0.51). The TOL had no strong correlation with the two WHODAS subscales or with employment status. Both the WHODAS-DF and the WHODAS-SF showed low correlations with Employment status (*r* = 0.17; *r* = 0.23).

**Table 2:** Correlation matrix.

	*N*	*1*	2	3	4	5	6	7
*1. Age*	309							
*2. Sex*	308	**0.13**						
*3. BRIEF-A (Z-score)*	266	0.03	0.05					
*4. TOL (Z-score)*	270	**−0.15**	−0.09	−0.04				
*5. WHODAS – DF*	282	**0.15**	**0.14**	**0.56**	−0.10			
*6. WHODAS – SF*	254	**0.16**	**0.14**	**0.51**	0.01	**0.67**		
*7. Employment status*	283	**0.25**	0.05	0.10	−0.09	**0.17**	**0.23**	

**Bold** = significant correlation on *p* = .05.

BRIEF-A = Behavioural Rating Inventory of Executive Functioning – Adults; TOL = Tower of London; WHODAS-DF = World Health Organization – Disability Assessment Schedule – Daily Functioning; WHODAS-SF = World Health Organization – Disability Assessment Schedule – Social Functioning.

**Fig. 2:**
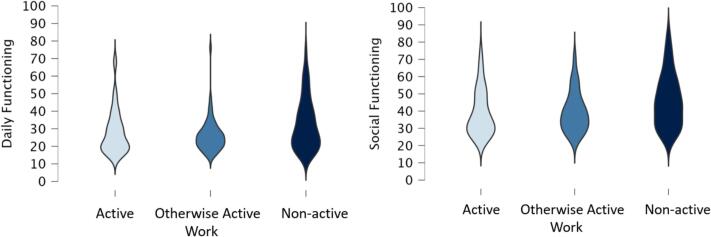
Violin plots of active, otherwise active and non-active groups on daily and social functioning.

We also analysed the WHODAS scores per employment status using violin plots (see Fig. 2). This was done based on visual inspection. The perceived disabilities were notably higher for Social Functioning than for Daily Functioning. This means there were more perceived disabilities for the WHODAS-SF than for the WHODAS-DF. The dispersion of perceived disabilities for the Active and Otherwise Active groups was higher for Social Functioning than for Daily Functioning, meaning there were more perceived disabilities for these two groups on the WHODAS Social Functioning scale than the Daily Functioning scale. The Non-active group showed similar dispersions for both the WHODAS-DF and WHODAS-SF.

### 3.3 Regression models

Table 3 shows the results of the regression analysis for daily functioning and Table 4 the results of the regression analysis for social functioning. Likelihood-ratio tests showed that adding interaction terms did not improve model fit: LR = 2*(LnL1−LnL2). For the WHODAS-DF models this resulted in LR = 2*(Ln(85.591) - Ln(92.662)), which resulted in LR = 2*(4.5290–4.4533) = 0.15 (df = 5, *p* = .99). For the WHODAS-SF models this resulted in LR = 2*(Ln(90.839)- Ln(83.665)), which resulted in LR = 2*(4.5091–4.4268) = 0.17 (df = 5, p = .99). Therefore, the models without the interactions were further interpreted.

**Table 3:** Regression models of executive functioning on daily functioning (WHO-DAS DF).

	Model 1		Model 2	
Likelihood Ratio Chi-square	92.662		85.591	
Parameter	β (SE)	Wald X^2^	β (SE)	Wald X^2^
*Intercept*	29.5 (2.6)	133.2***	29.2 (2.6)	128.3***
*Sex (male)*	−4.6 (1.5)	9.6**	−4.4 (1.5)	8.8**
*Age (centred)*	0.07 (0.1)	1.3	0.07 (0.6)	1.3
*PANSS total score*	2.2 (1.0)	4.8*	2.2 (1.0)	4.9*
*BRIEF-A (Z-score)*	4.8 (0.9)	22.8***	5.1 (0.7)	49.8***
*TOL (Z-score)*	−2.4 (1.0)	5.3*	−1.8 (0.7)	6.0*
*Employment – 3. Non-active*				
*Employment – 2. Otherwise Active*	−4.6 (1.8)	7.0**	−4.5 (1.8)	6.5*
*Employment – 1. Active*	−0.5 (1.8)	0.1	−1.0 (1.8)	0.3
BRIEF-A (Z-score) * TOL (Z-score)	0.7 (0.8)	0.9		
Employment – 3. Non-active * BRIEF-A (Z-score)				
Employment – 2. Otherwise Active * BRIEF-A (Z-score)	−0.7 (1.8)	0.2		
Employment – 1. Active * BRIEF-A (Z-score)	2.9 (1.6)	3.2		
Employment – 3. Non-active * TOL (Z-score)				
Employment – 2. Otherwise Active * TOL (Z-score)	1.7 (1.6)	1.2		
Employment – 1. Active * TOL (Z-score)	−0.2 (1.9)	0.0		

Model 1 = GLM model with interactions, Model 2 = GLM model without interactions. Wald-test *p* < .05*, *p* < .01**, *p* < .001***.

BRIEF-A = Behavioural Rating Inventory of Executive Functioning – Adults; TOL = Tower of London; WHODAS-DF = World Health Organization – Disability Assessment Schedule – Daily Functioning; WHODAS-SF = World Health Organization – Disability Assessment Schedule – Social Functioning.

**Table 4:** *Regression models of executive functioning on social functioning* (WHO-DAS SF).

	Model 1		Model 2	
Likelihood Ratio Chi-square	83.665		90.839	
Parameter	β (SE)	Wald X^2^	β (SE)	Wald X^2^
*Intercept*	37.6 (2.8)	180.0***	37.6 (2.8)	174.6***
*Sex (male)*	−5.2 (1.7)	10.0**	−5.2 (1.7)	9.8**
*Age (centred)*	0.0 (0.1)	0.1	0.0 (0.1)	0.1
*PANSS total score*	4.8 (1.1)	18.1***	4.8 (1.1)	17.5***
*BRIEF-A (Z-score)*	4.6 (1.1)	18.0***	5.1 (0.8)	38.8***
*TOL (Z-score)*	−1.1 (1.2)	1.0	−1.1 (0.8)	0.0
*Employment – 3. Non-active*				
*Employment – 2. Otherwise Active*	−3.3 (1.9)	2.9	−3.2 (2.0)	2.7
*Employment – 1. Active*	−1.6 (2.1)	0.6	−2.3 (2.0)	1.2
BRIEF-A (Z-score) * TOL (Z-score)	0.7 (0.8)	0.8		
Employment – 3. Non-active * BRIEF-A (Z-score)				
Employment – 2. Otherwise Active * BRIEF-A (Z-score)	−0.1 (2.1)	0.0		
Employment – 1. Active * BRIEF-A (Z-score)	2.0 (1.8)	1.2		
Employment – 3. Non-active * TOL (Z-score)				
Employment – 2. Otherwise Active * TOL (Z-score)	3.2 (1.7)	3.5		
Employment – 1. Active * TOL (Z-score)	−0.6 (2.1)	0.1		

Model 1 = GLM model with interactions, Model 2 = GLM model without interactions. * Wald-test *p* < .05; * *p* < .01; ** *p* < .001***.

BRIEF-A = Behavioural Rating Inventory of Executive Functioning – Adults; TOL = Tower of London; WHODAS-DF = World Health Organization – Disability Assessment Schedule – Daily Functioning; WHODAS-SF = World Health Organization – Disability Assessment Schedule – Social Functioning.

These simpler models without interactions showed that the BRIEF-A had an effect on both the WHODAS-DF (β = 5.1, SE = 0.7) and on the WHODAS-SF (β = 5.1, SE = 0.8), meaning worse scores on the BRIEF-A resulted in more perceived disabilities on both the WHODAS-DF and WHODAS-SF. The TOL had an effect only on the WHODAS-DF (β = −1.8, SE = 0.7)). For employment status, being Otherwise Active was associated with lower scores (i.e. less perceived disabilities) on the WHODAS-DF than being Non-active was (β = −4.5, SE = 1.8).

Assumptions check for both the models without interactions, showed that both the WHODAS-DF and WHODAS-SF were somewhat skewed to the left (i.e. mainly lower perceived disabilities scores). However, this skewness was only minor, and all other assumptions checks were within limits.

## 4 Discussion

This study investigated the association between functional recovery (operationalized as performance-based EF and self-report EF) and social recovery (operationalized as Daily Functioning and Social Functioning). The findings of this cohort align with expectations based on comparable studies. The PANSS-R scores indicate a relatively low overall symptom burden within this sample. Additionally, the BRIEF-A scores in this study were higher than those observed in similar patient groups but remained lower than those of healthy controls (Bulzacka et al., 2013). Performance on the TOL was comparable to that of other outpatient populations (Wang et al., 2016; Segarra et al., 2011). The WHODAS scores revealed a greater number of perceived disabilities in the Social Functioning domain compared to the Daily Functioning domain. As anticipated, across all WHODAS domains, our study population exhibited lower scores than a younger patient cohort (Guilera et al., 2012). We hypothesized that better performance-based executive functioning would be associated with less disabilities in Daily Functioning, while self-report executive functioning would be associated with better Social Functioning. These hypotheses were partly confirmed: whereas self-report EF had a large association with both Daily Functioning and Social Functioning, performance-based EF had only a small association with Daily Functioning. We also hypothesized that an Active employment status would interact with executive functions and improve social recovery more than in clients who were Non-active or Otherwise Active. However, we found no interaction effect. Interestingly, there was a small difference on the Daily Functioning scale for the Active group compared to the Non-active group, but not for the Social Functioning scale.

The differences in the associations of social functioning with self-report and performance-based EF underlines the importance of measuring EF in complimentary ways. Although the difference may be due to validity problems of either measure (Gioia et al., 2010; van Aken et al., 2023), it is possible that both measures reflect a different aspect of executive functions (Nordvall et al., 2017; van Aken et al., 2023; Ten Eycke and Dewey, 2016). Recent research in ADHD suggests that performance-based measures test a person's ability, such as their skill or talent, whereas self-report measures test their application of that ability in an environmental context (Bowie and Harvey, 2006; Weyandt et al., 2014). This might imply that a greater ability to use your skills in an environmental context reduces the number of disabilities perceived in Daily and Social Functioning. However, the possession of skills influences only the limitations perceived in Daily Functioning assessed on the WHODAS, a fact that is supported by the administrative characteristics of performance-based tasks. Performance-based measures are administered in an environment with little or no distractions. For Daily Functioning, daily tasks are often more easily manageable and subject to less pressure from the environment. In the case of our patient group, more than half are single and live alone, meaning they often do their daily chores without many distractions. Furthermore, self-report measures are known to be influenced by emotional state (Kajonius and Björkman, 2020; Buchanan, 2016). It might be that the association between the self-report measure of executive function, as well as the self-reported perceived disabilities on the WHODAS are influenced by emotional state in the same manner. Moreover, self-report may more adequately capture actual impairments in daily life. This should be investigated further.

There was also a difference regarding employment status and Daily and Social Functioning. The Otherwise Active group experienced fewer limitations on their Daily Functioning than the Active and Non-active groups. This was unexpected, as previous studies found that the Active group experiences less disability than the unemployed group (Chen et al., 2020; Johnson et al., 2014). In our study, the disability scores for Daily Functioning did not differ between the Active and Non-active group. The Otherwise Active group scored better, which may be explained by the fact that the Otherwise Active group consists mainly of people who act as volunteers – probably a less demanding environment, or even one that is adjusted to the clients' skills and thus has a better person-environment fit. A paid job, on the other hand, may be experienced as highly demanding, with regard both to time and the organizational skills required. It is also the case that, unlike paid work, volunteering is often scheduled for fewer days per week, usually only one or two. A further factor is that working in a paid job is more likely to require a person to commute to work. Due to a longer commute and a longer working week, the Active group might thus have less time to do their daily tasks, possibly resulting in their experiencing more difficulties with Daily Functioning. Lastly, the Otherwise Active group also includes people who are not currently employed but are actively looking for paid work. They might feel comfortable looking for a job because they currently experience fewer limitations, which allows them to get back into the game. As for the lack of a difference between the Non-active and Active groups, it is possible that, even without work, those in the Non-active group experience their environment as being as demanding as those in Active group experience *their* environment *with* work.

To summarise, this study has a few important clinical implications. First, it shows the importance of measuring executive functions using both a performance-based and self-report measure. Both show different impairments for patients and give different directions for care: impairments in performance-based measures are thought to reflect the possession of skills. Clinically, focus should then be on learning the skill. If impairments in self-report measures are shown, however, this is thought the reflect impairments in the use of these skills in a daily context. Treatment should then focus on contextual learning and the application of the skills under duress, for example. Second, when looking at daily activities like work or studies, treatment goals should focus on attaining a good person-environment fit, especially when there are perceived disabilities.

## 5 Strengths and limitations

To the best of our knowledge, this is the first study to investigate the relationship between functional and social recovery in a group of patients with a psychotic disorder who have been in care for a longer period. Much of the earlier research on functional and social recovery was conducted in First-Episode Psychosis (FEP) patient groups. As FEP patients have had fewer psychotic episodes than our chronic psychotic patients, they may have better rates of functional and social recovery that are not generalizable to our own group (Robinson et al., 2004; Van Duin et al., 2021; Karambelas et al., 2018). We therefore examined the association between functional and social recovery in a broad psychosis population. As expected, we found that functional and social recovery were even lower in our cohort than in these earlier studies.

Demographic variables show that this cohort is representative of patients in Dutch Ambulatory Mental Health care (Kortrijk et al., 2019a). For example, employment rates in this cohort are about 25 %, similar to the rates found in previous studies based on ROM data (Kortrijk et al., 2019b).

We were able to include three employment status groups that were representative of the FACT population (Kortrijk et al., 2019b): Active, Otherwise Active and Non-active. This enabled us to look analyse the influence of employment status on perceived disabilities. Earlier studies that examined disabilities took account only of having work ‘yes or no’.

This study has three main limitations. First, we used a two-way division of the WHODAS 2.0, which turned out not to have a very good model fit. Both subscales also seemed to overlap and were moderately correlated. This raises the question if we measure two different constructs, or whether the overlap between the two constructs might be large, resulting in the poor model fit and moderate correlation.

For the Non-active group, we did not distinguish between people who were not able to work, willing to work, or enjoying early retirement. The disabilities may have differed between these three groups. For example, whereas those in early retirement may have retired simply because it was possible and felt good after many years of work, those not looking for a job did not look because of the disabilities they perceived.

Secondly, as disability is known to be influenced by emotional state (Kajonius and Björkman, 2020; Buchanan, 2016), we should be aware that self-report measurements can be influenced by emotional state or insight (Meltzer et al., 2017; Galliot et al., 2022; Shwartz et al., 2020), possibly leading to more overlap between the self-report measures and the scores for the disability people experienced, As it is therefore possible that the association we found between the BRIEF-A and the WHODAS disability measures was moderated by, or even reflective of, emotional state, future research should examine the links between emotional state and both methods of measuring executive functioning.

Finally, we took no account of medication or physical health, even though both are important factors with a direct influence on disability scores. Since weight gain, parkinsonism and drowsiness are common side effects of anti-psychotic medication, these factors may even interact, influencing the perceived disability, which, combined with cognitive deficits, might even be worse (ÜÇOk and Gaebel, 2008).

### 5.1 Future research

As this study has a cross-sectional design, a logical next step would be to investigate the relationship between functional and social recovery over time. This might make it possible to identify any latency effect between having a job and improvements in overall disabilities. Since baseline measurements in this cohort are taken at any point in the patients' treatment, account should also be taken of how long individual clients have been in care and the nature of their treatment so far. Rather than the two subscales we used, it would also be interesting to use all six domains of the WHODAS separately. By providing a more detailed understanding of the relationship between functional and social recovery, this might lead to more personalized treatment.

## 6 Conclusion

Self-report and performance-based measures for executive functioning have different associations with Daily and Social Functioning. While both measures are related to disabilities in Daily Functioning, only self-report measures are related to disabilities in Social Functioning. Meanwhile, being Otherwise Active – i.e., being a volunteer or looking for a job – also has an association with disabilities in Daily Functioning. As well as measuring self-reported executive functioning, clinicians should also consider employment status when trying to treat their clients' perceived disabilities.

## CRediT authorship contribution statement

**B.C. van Aken:** Writing – review & editing, Writing – original draft, Software, Resources, Project administration, Methodology, Formal analysis, Data curation, Conceptualization. **R. Rietveld:** Writing – review & editing, Writing – original draft, Project administration, Methodology, Investigation, Formal analysis, Data curation, Conceptualization. **A.I. Wierdsma:** Writing – review & editing, Methodology, Investigation, Formal analysis, Conceptualization. **Y. Voskes:** Writing – review & editing, Investigation, Conceptualization. **G.H.M. Pijnenborg:** Writing – review & editing, Supervision, Conceptualization. **J. van Weeghel:** Writing – review & editing, Supervision, Resources, Funding acquisition, Conceptualization. **C.L. Mulder:** Writing – review & editing, Validation, Supervision, Resources, Funding acquisition, Conceptualization.

## Declaration of competing interest

The authors have no conflicts of interest to declare.
