# Corrigendum to “A cross-sectional study on the association between executive functions and social disabilities in people with a psychotic disorder” [Schizophr. Res. Cogn. Volume 40 (2025), article number: 100349]

**DOI:** 10.1016/j.scog.2025.100360

**Published:** 2025-04-03

**Authors:** B.C. van Aken, R. Rietveld, A.I. Wierdsma, Y. Voskes, G.H.M. Pijnenborg, J. van Weeghel, C.L. Mulder

**Affiliations:** a Department of Psychiatry, Erasmus MC, Epidemiological and Social Psychiatric Research Institute, Rotterdam, the Netherlands; b Fivoor Forensic Psychiatric Institute, Rotterdam, the Netherlands; c Department of Ethics, Law and Humanities, Amsterdam UMC, Amsterdam, the Netherlands; d GGz Breburg, Tilburg, the Netherlands; e Department of Psychotic Disorders, GGZ, Drenthe, Assen, the Netherlands; f Department of Clinical and Developmental Neuropsychology, Faculty of Behavioural and Social Sciences, University of Groningen, Groningen, the Netherlands; g Phrenos Centre of Expertise, Utrecht, the Netherlands; h Tranzo Department, Tilburg School of Behavioural and Social Sciences, Tilburg University, Tilburg, the Netherlands; i Bavo-Europoort Mental Health Care, Rotterdam, the Netherlands

The authors regret <not stating clearly that B.C. van Aken & R. Rietveld contributed equally to the manuscript. We would like to add a corrigendum where it states that the first and second author (B.C. van Aken & R. Rietveld) contributed equally>.

The authors would like to apologise for any inconvenience caused.

